# Metachronous severe multisystem immune-related adverse events during tremelimumab plus durvalumab therapy for hepatocellular carcinoma: a clinicopathological autopsy case report

**DOI:** 10.3389/fonc.2026.1830325

**Published:** 2026-08-05

**Authors:** Takuto Nosaka, Junki Yamashita, Yu Akazawa, Tomoko Tanaka, Kazuto Takahashi, Tatsushi Naito, Masahiro Ohtani, Yukihiro Kimura, Yoshiaki Imamura, Hiroshi Tada, Motohiro Kobayashi, Yasunari Nakamoto

**Affiliations:** 1 Department of Gastroenterology, Faculty of Medical Sciences, University of Fukui, Fukui, Japan; 2 Department of Otorhinolaryngology-Head and Neck Surgery, Faculty of Medical Sciences, University of Fukui, Fukui, Japan; 3 Division of Diagnostic Pathology/Surgical Pathology, University of Fukui Hospital, Fukui, Japan; 4 Department of Cardiovascular Medicine, Faculty of Medical Sciences, University of Fukui, Fukui, Japan; 5 Department of Pathology, Faculty of Medical Sciences, University of Fukui, Fukui, Japan

**Keywords:** autopsy, hepatocellular carcinoma, immune-related adverse events, myocarditis, tremelimumab plus durvalumab

## Abstract

Immune checkpoint inhibitors (ICIs) have improved outcomes in advanced hepatocellular carcinoma (HCC), but severe multisystem immune-related adverse events (irAEs) remain a major clinical challenge. We report an autopsy-confirmed case of advanced HCC treated with tremelimumab plus durvalumab that developed severe metachronous multisystem irAEs characterized by sequential pancreatitis, laryngeal edema with sialadenitis, and myocarditis. Twelve days after treatment initiation, the patient presented with fever and abdominal distension, and computed tomography showed CTCAE grade 3 pancreatitis. On hospital day 6, he developed life-threatening laryngeal edema requiring intubation and steroid pulse therapy. Although the airway edema improved, complete atrioventricular block developed on day 23. Cardiac troponin I increased to 42,006 pg/mL, and endomyocardial biopsy demonstrated CD8-predominant myocarditis consistent with CTCAE grade 4 irAE. Cardiac conduction recovered after additional corticosteroid therapy, but the patient subsequently died of septic shock. Autopsy revealed CD8-positive lymphocytic infiltration not only in clinically recognized irAE-affected organs, including the pancreas, salivary glands, pharynx/larynx, and heart, but also in clinically silent organs, including the kidneys and intestine, indicating widespread subclinical immune-mediated injury. Multiplex immunofluorescence analysis demonstrated CD3-positive T-cell infiltration enriched for CD8-positive T cells, with variable or focal Granzyme B expression across affected tissues. In contrast, CD68-positive macrophages and CD66b-positive neutrophils were not the dominant inflammatory populations. This clinicopathological autopsy case supports the concept that clinically apparent irAEs may represent only the visible portion of systemic CD8-positive T-cell–dominant immune-mediated injury. Severe irAEs after tremelimumab plus durvalumab therapy may progress metachronously and involve clinically silent organs beyond those recognized during life, highlighting the need for continued multisystem monitoring after the onset of severe irAEs.

## 1 Introduction

The introduction of immune checkpoint inhibitors (ICIs) has substantially changed the systemic treatment landscape for advanced hepatocellular carcinoma (HCC) (1). In particular, the combination of the anti–programmed death-ligand 1 (PD-L1) antibody durvalumab and the anti–cytotoxic T-lymphocyte–associated protein 4 (CTLA-4) antibody tremelimumab (the STRIDE regimen) demonstrated clinical efficacy in the HIMALAYA trial and has become an established treatment option for advanced HCC (2).

ICIs can induce a broad spectrum of immune-related adverse events (irAEs) through immune dysregulation (3). Although many irAEs are manageable, some involve life-threatening toxicities such as myocarditis, neurologic complications, or severe multiorgan inflammation (4). In addition, irAEs may arise not only simultaneously but also sequentially across different organs, making diagnosis and management more challenging (3, 5). Moreover, the full extent of systemic immune-mediated injury may be underestimated when evaluation is based only on clinically apparent organ involvement. Autopsy studies have suggested that clinically overt irAEs may represent only a fraction of immune-related organ pathology, with inflammatory changes also occurring in organs that were not clinically recognized as irAE targets during life (6, 7). Therefore, clinicopathological correlation using biopsy and autopsy specimens may provide important insight into the systemic nature of ICI-associated immune injury. In particular, multiplex immunofluorescence analysis of inflammatory cell composition may help distinguish T-cell–mediated immune injury from infectious inflammation or nonspecific terminal inflammatory changes.

Here, we report a clinicopathological autopsy case of advanced HCC treated with tremelimumab plus durvalumab that developed severe metachronous multisystem irAEs, including pancreatitis, laryngeal edema with sialadenitis, and myocarditis. The case is notable for sequential progression despite corticosteroid therapy, biopsy-proven CD8-predominant myocarditis, and autopsy evidence of CD8-positive T-cell infiltration in both clinically recognized and clinically silent organs. We further performed multiplex immunofluorescence analysis to characterize the inflammatory infiltrates and to evaluate whether the multiorgan lesions represented systemic T-cell–mediated immune injury rather than macrophage-dominant nonspecific inflammation or neutrophil-dominant infectious inflammation.

## 2 Case description

A 65-year-old man was admitted with fever and abdominal distension. Five years earlier, he had been diagnosed with hepatocellular carcinoma (HCC) arising in the setting of alcohol-related liver cirrhosis and had undergone transarterial chemoembolization and radiofrequency ablation. One year before the current admission, computed tomography (CT) revealed multifocal HCC involving both hepatic lobes, and lenvatinib was initiated. Because of radiologic disease progression, tremelimumab plus durvalumab was started 12 days before admission (Day −12).

**Figure 1:**
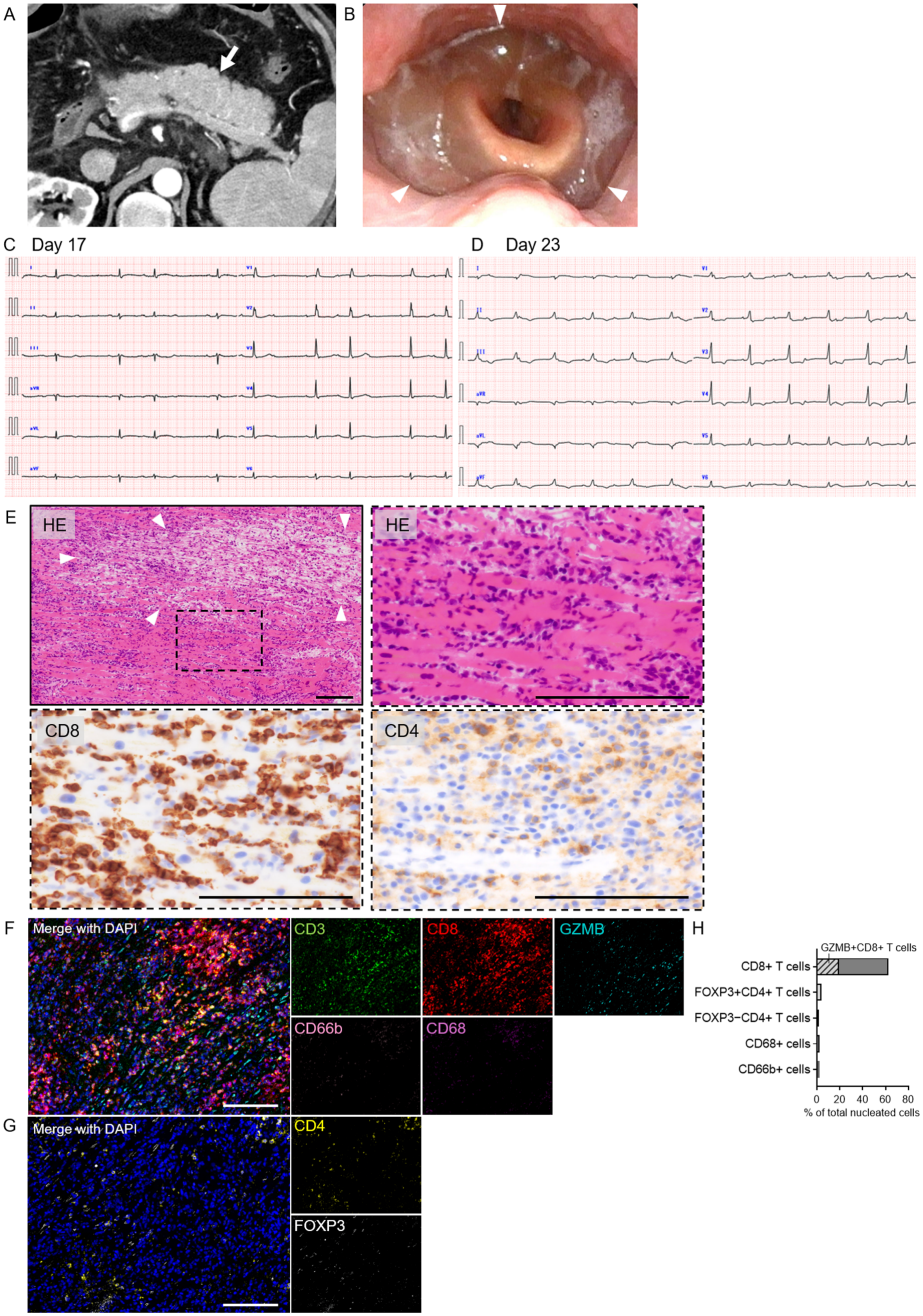
Clinicopathological findings of multiorgan immune-related adverse events. **(A)** Early-phase contrast-enhanced abdominal computed tomography **(CT)** showing pancreatic enlargement (white arrow). **(B)** Laryngoscopic findings demonstrate severe laryngeal edema with upper airway narrowing (white arrowhead). **(C, D)** Twelve-lead electrocardiograms. A Wenckebach-type second-degree atrioventricular block was noted on hospital day 17 **(C)**, which progressed to complete atrioventricular block on hospital day 23 **(D)**. **(E)** Hematoxylin and eosin–stained sections of a right ventricular endomyocardial biopsy specimen and immunohistochemical staining for CD8 and CD4, revealing marked lymphocytic and plasma cell infiltration with focal myocardial dropout (white arrowhead). **(F)** Multiplex immunofluorescence staining of the right ventricular endomyocardial biopsy specimen for DAPI, CD3, CD8, CD4, Granzyme B, CD66b, and CD68. **(G)** FOXP3 multiplex immunofluorescence panel of the right ventricular endomyocardial biopsy specimen for DAPI, CD4, and FOXP3. Scale bar: 100 µm. **(H)** Quantitative analysis of immune-cell subsets in the right ventricular endomyocardial biopsy specimen. CD8-positive T cells were defined as CD3-positive CD8-positive cells. CD8-positive Granzyme B-positive cells and CD8-positive Granzyme B-negative cells were separately quantified to evaluate variable cytotoxic activation among CD8-positive T cells. FOXP3-positive CD4-positive cells were defined as CD4-positive FOXP3-positive cells in the FOXP3 panel. CD68-positive cells and CD66b-positive cells were interpreted as macrophage-lineage cells and neutrophils, respectively. Percentages indicate the proportion of each immune-cell subset among total nucleated cells in the analyzed region.

On admission, his temperature was 37.9 °C, and abdominal CT demonstrated pancreatic enlargement with inflammatory changes extending below the inferior renal poles, consistent with acute pancreatitis (**Figure 1A**). Laboratory evaluation showed elevated pancreatic enzymes and inflammatory markers (**Table 1**). Differential diagnoses for acute pancreatitis included biliary pancreatitis, biliary obstruction, pancreatic tumor involvement, alcohol-related pancreatitis, infectious pancreatitis, and pancreatitis associated with other drugs. Imaging showed no apparent biliary obstruction or pancreatic tumor involvement, and there was no clear clinical evidence of infection or alcohol-related acute pancreatitis. Given the close temporal relationship to tremelimumab plus durvalumab initiation, the patient was diagnosed with Common Terminology Criteria for Adverse Events (CTCAE) grade 3 irAE-associated pancreatitis. Conservative treatment resulted in improvement of abdominal symptoms and hyperamylasemia.

**Table 1:** Laboratory examinations.

WBCs	5,400	/µL	Aspartate aminotransferase	40	U/L
RBCs	414×10^4^	/µL	Alanine aminotransferase	26	U/L
Hemoglobin	11.0	g/dL	LDH	321	U/L
Hematocrit	36.0	%	ALP	110	U/L
Platelets	14.7×10^4^	/µL	GGT	122	U/L
			Total bilirubin	1.2	mg/dL
PT-INR	1.36		Total protein	6.1	g/dL
APTT	44.3	sec	Albumin	2.9	g/dL
Fibrinogen	462	mg/dL	BUN	17	mg/dL
FDP	25.5	μg/mL	Creatinine	0.97	mg/dL
D-dimer	9.5	μg/mL	Total amylase	1191	U/L
			Pancreatic amylase	1160	U/L
CEA	2.5	ng/mL	CRP	8.23	mg/dL
CA19-9	36.5	U/mL	HbA1c	5.8	%
AFP	2.8	ng/mL			
DCP	125	mAU/mL			

WBCs white blood cells, RBCs red blood cells, PT-INR prothrombin time-international normalized ratio, APTT activated partial thromboplastin time, FDP fibrinogen/fibrin degradation products, CEA carcinoembryonic antigen, CA19–9 cancer antigen 19–9, AFP alpha-fetoprotein, DCP des-gamma-carboxy prothrombin, LDH lactate dehydrogenase, ALP alkaline phosphatase, GGT gamma-glutamyl transpeptidase, BUN blood urea nitrogen, CRP C-reactive protein, HbA1c glycated hemoglobin.

However, on hospital day 6, he newly developed dyspnea and dysphagia. Laryngoscopy and CT revealed severe laryngeal edema causing upper-airway narrowing together with salivary gland enlargement (**Figure 1B**). Differential diagnoses included infectious laryngitis or epiglottitis, allergic angioedema, tumor-related airway obstruction, radiation-related changes, and drug-induced edema. The concomitant salivary gland enlargement, absence of clear local infection or tumor obstruction, and temporal association with immune checkpoint inhibitor therapy supported immune-related laryngeal edema and sialadenitis. Laryngeal edema with severe upper-airway stenosis and concomitant sialadenitis was diagnosed as CTCAE grade 4 irAEs, and steroid pulse therapy (methylprednisolone 1 g/day for 3 days) was initiated under endotracheal intubation. The laryngeal edema gradually improved, and the patient was extubated on day 16.

**Figure 2:**
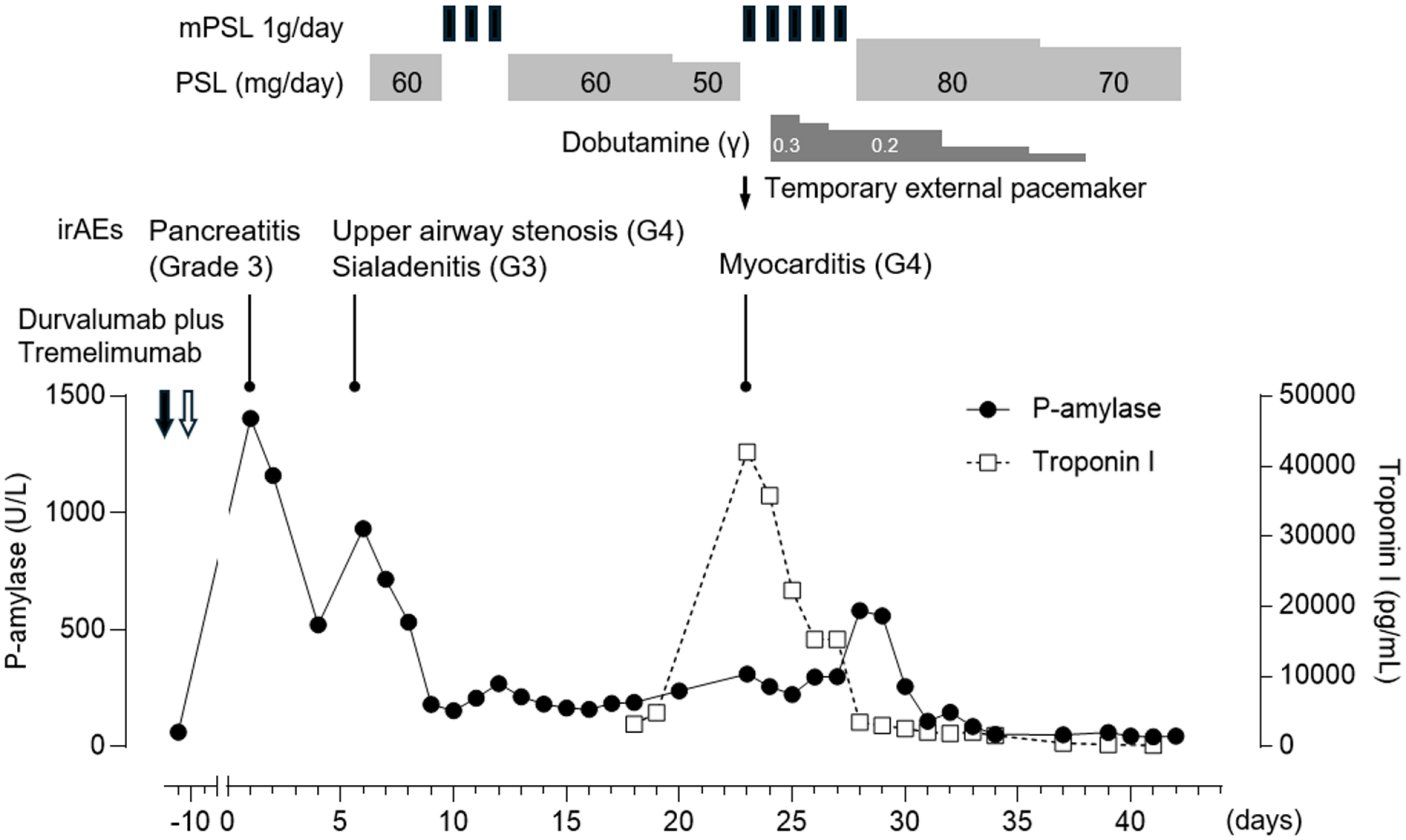
Clinical timeline of metachronous multisystem immune-related adverse events after tremelimumab plus durvalumab therapy. The timeline shows administration of tremelimumab plus durvalumab, onset of pancreatitis, development of laryngeal edema with sialadenitis, corticosteroid pulse therapy, extubation, onset of Wenckebach-type atrioventricular block, progression to complete atrioventricular block, endomyocardial biopsy, temporary pacemaker placement and removal, septic shock, and death. Serial changes in pancreatic amylase and cardiac troponin I are shown to illustrate the metachronous progression of organ-specific irAEs. irAE, immune-related adverse event; mPSL, methylprednisolone; PSL, prednisolone.

Subsequently, while receiving oral prednisolone (50 mg/day), electrocardiography on day 17 revealed a Wenckebach-type second-degree atrioventricular block (**Figure 1C**), which progressed to complete atrioventricular block by day 23 (**Figure 1D**). Laboratory evaluation demonstrated marked elevation of cardiac troponin I (42,006 pg/mL), and the left ventricular ejection fraction decreased from 60% to 40% (**Figure 2**). Differential diagnoses for myocarditis included acute coronary syndrome, Takotsubo cardiomyopathy, sepsis-related cardiomyopathy, electrolyte abnormalities, viral myocarditis, and other drug-induced cardiotoxicity. The new-onset atrioventricular conduction disturbance, marked troponin I elevation, reduced left ventricular ejection fraction, and the close temporal association with immune checkpoint inhibitor therapy strongly suggested immune checkpoint inhibitor–associated myocarditis (CTCAE grade 4). A right ventricular endomyocardial biopsy was therefore performed, and a temporary transvenous pacemaker was placed. Histopathological examination demonstrated myocyte injury with inflammatory cell infiltration predominantly composed of CD8(+) T lymphocytes (**Figure 1E**). Following a second course of steroid pulse therapy (methylprednisolone 1 g/day for 5 days), cardiac troponin I levels decreased, and both atrioventricular conduction and left ventricular ejection fraction improved, allowing removal of the temporary pacemaker on day 38.

**Figure 3:**
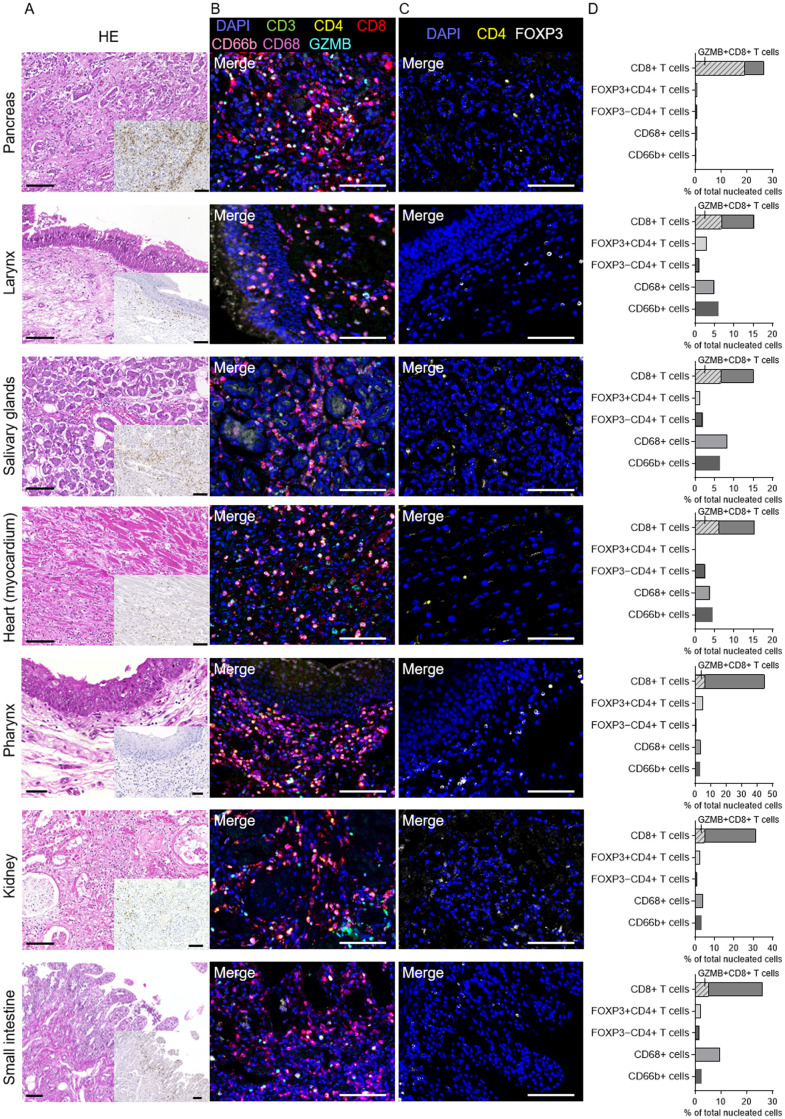
Organ-wide histopathological and multiplex immunofluorescence findings of systemic immune-mediated injury. Representative findings are shown for seven organs or tissue sites examined at autopsy. **(A)** Hematoxylin and eosin staining demonstrates tissue injury and inflammatory cell infiltration in each organ. The inset in the lower right shows CD8 immunohistochemistry. Scale bar: 100 µm. **(B)** Multiplex immunofluorescence staining using the Opal panel, including DAPI, CD3, CD4, CD8, Granzyme B, CD68, and CD66b, was performed to characterize the inflammatory cell composition. **(C)** Multiplex immunofluorescence staining using the FOXP3 Opal panel, including DAPI, CD4, and FOXP3, was performed to evaluate the FOXP3-positive CD4-positive cell component. Scale bar: 100 µm. **(D)** Quantitative analysis of immune-cell subsets in representative organs and tissue sites. CD8-positive T cells were defined as CD3-positive CD8-positive cells. CD8-positive Granzyme B-positive cells and CD8-positive Granzyme B-negative cells were separately quantified to evaluate variable cytotoxic activation among CD8-positive T cells. FOXP3-positive CD4-positive cells were defined as CD4-positive FOXP3-positive cells in the FOXP3 panel. CD68-positive cells and CD66b-positive cells were interpreted as macrophage-lineage cells and neutrophils, respectively. Percentages indicate the proportion of each immune-cell subset among total nucleated cells in the analyzed regions.

Later, on day 41, the patient developed septic shock secondary to cellulitis of the right lower extremity and catheter-related infection and died on day 42. Autopsy revealed immune-mediated pathological findings not only in clinically recognized irAE-affected organs, including the pancreas, salivary glands, pharynx/larynx, and heart, but also in clinically silent organs such as the kidneys and intestine (**Figure 3**; **Supplementary Table 1**). The submandibular gland showed periductal fibrosis and lymphocytic infiltration with CD8-positive cells in the glandular parenchyma and ductal epithelium. The heart showed patchy fibrosis and lymphocytic infiltration throughout the ventricular walls, consistent with the CD8-predominant myocarditis diagnosed by endomyocardial biopsy. The pancreas showed peripancreatic fat necrosis, lobular fibrosis, acinar atrophy, and CD8-positive lymphocytic infiltration. The hypopharynx and intestine showed CD8-positive lymphocytic infiltration in the subepithelial stroma, lamina propria, and focally within the epithelium. In the kidneys, acute tubular necrosis was present, likely reflecting terminal circulatory failure; however, CD8-positive lymphocytes were also observed in the interstitium and tubular epithelium, suggesting a concomitant immune-mediated tubulointerstitial component. Bone marrow examination showed a generally preserved number of megakaryocytes, suggesting possible hematologic irAE involvement in the thrombocytopenia, although the precise hematologic phenotype should be interpreted cautiously. Although terminal septic shock and circulatory failure contributed to some findings, including hepatic congestion and acute tubular necrosis, the organ-based CD8-positive lymphocytic infiltration supported widespread immune-mediated tissue injury. Additional multiplex immunofluorescence staining for CD3, CD4, CD8, Granzyme B, CD68, CD66b, and DAPI was performed using the endomyocardial biopsy specimen and representative autopsy tissues (**Figures 1F, G**, **Figure 3**; **Supplementary Figures 1**, **2**). Quantitative immune-cell analysis was performed for the endomyocardial biopsy specimen and representative organs or tissue sites (**Figures 1H**, **3D**). In the endomyocardial biopsy specimen, the inflammatory infiltrates were predominantly composed of CD3-positive T cells, most of which were CD8-positive. A subset of CD8-positive cells expressed Granzyme B, supporting cytotoxic T-cell activation. Quantitative analysis showed that CD8-positive T cells constituted a substantial proportion of total nucleated cells in the analyzed regions of irAE-associated organs and tissue sites, including the endomyocardial biopsy specimen (62.7%), autopsy myocardium (15.4%), pancreas (26.7%), salivary gland (15.1%), larynx (15.3%), pharynx (45.1%), kidney (31.4%), and small intestine (26.2%). In contrast, CD8-positive T-cell infiltration was limited in the sampled hepatic tumor and background liver, accounting for 1.8% and 5.4% of total nucleated cells, respectively. CD68-positive macrophages were scattered, and CD66b-positive neutrophils were not the dominant inflammatory populations in the irAE-associated lesions. Granzyme B expression among CD8-positive T cells varied among organs; CD8-positive Granzyme B-positive cells were present in several lesions, whereas CD8-positive Granzyme B-negative cells were the major CD8-positive subset in some organs, including the pharynx, kidney, and small intestine. FOXP3 mini-panel staining showed that FOXP3-positive CD4-positive cells represented only a small subset and were relatively sparse compared with the prominent CD8-positive T-cell infiltration. Together, these findings supported systemic CD8-positive T-cell–dominant immune-mediated injury with variable cytotoxic activation, rather than macrophage-dominant nonspecific inflammation, neutrophil-dominant infectious inflammation, or Treg-rich inflammation.

## 3 Discussion

This case illustrates the metachronous progression of severe irAEs, including pancreatitis, laryngeal edema with sialadenitis, and myocarditis, after tremelimumab plus durvalumab therapy for HCC. The most important lesson from this case is that clinically apparent irAEs may not fully reflect the extent of systemic immune-mediated organ injury. Although pancreatitis, sialadenitis, upper-airway involvement, and myocarditis were clinically recognized during life, autopsy revealed CD8-positive T-cell infiltration also in clinically silent organs, including the kidney and intestine. Thus, clinically visible irAEs may represent only the visible portion of systemic immune-mediated injury.

The STRIDE regimen combines a single priming dose of the anti-CTLA-4 antibody tremelimumab with the anti-PD-L1 antibody durvalumab and has demonstrated an overall survival benefit in unresectable HCC in the HIMALAYA trial (2). Subsequent analyses of the HIMALAYA study have also evaluated immune-mediated adverse events after tremelimumab plus durvalumab therapy, highlighting the clinical importance of recognizing and managing immune-mediated toxicity in patients with unresectable HCC (8). However, dual checkpoint blockade can enhance systemic T-cell immune responses and may increase the risk of severe immune-mediated toxicity. ICI-related irAEs can affect multiple organs, including the skin, gastrointestinal tract, liver, endocrine organs, lungs, kidneys, nervous system, and heart, and their timing may differ across organs (3, 9, 10). Therefore, organ injury after tremelimumab plus durvalumab therapy should not always be interpreted as an isolated organ-specific event, but may represent part of a broader systemic immune activation process.

Multisystem irAEs have been previously reported. Shankar et al. described multisystem irAEs associated with ICIs in patients with non-small cell lung cancer (5). However, many previous reports have been based mainly on clinically recognized combinations of irAEs, whereas systematic autopsy evaluation of clinically silent organ involvement remains limited. The present case differs from prior reports in several respects: pancreatitis, laryngeal edema with sialadenitis, and myocarditis developed metachronously after STRIDE therapy for HCC; myocarditis was confirmed during life by endomyocardial biopsy; and autopsy revealed CD8-positive T-cell infiltration in clinically silent organs, including the kidney and intestine. These features position this case as a clinicopathological autopsy report demonstrating a discrepancy between clinically apparent irAEs and occult systemic immune-mediated injury.

Previous autopsy studies also support the possibility that clinically apparent irAEs may underestimate the true extent of immune-related organ pathology. Koelzer et al. reported systemic inflammatory infiltration in multiple organs that had not been clinically recognized during life in a melanoma patient treated with ICIs (6). Similarly, Maccio et al. showed in a 13-year autopsy cohort that inflammatory changes could be identified in various organs after ICI therapy, including lesions that had not been diagnosed clinically (7). Consistent with these studies, our case showed CD8-positive T-cell infiltration in clinically silent organs, suggesting that ICI-induced immune activation may extend beyond clinically evident organ involvement.

Myocarditis was the most clinically severe irAE in the present case. ICI-associated myocarditis is rare but potentially fatal and may present with troponin elevation, conduction abnormalities, ventricular dysfunction, and malignant arrhythmias (11–13). In this case, a Wenckebach-type atrioventricular block progressed to complete atrioventricular block, cardiac troponin I increased markedly, and left ventricular ejection fraction decreased. Endomyocardial biopsy demonstrated myocyte injury with CD8-predominant lymphocytic infiltration. Furthermore, multiplex immunofluorescence of the endomyocardial biopsy specimen showed CD3-positive T-cell infiltration with CD8 enrichment and focal Granzyme B expression, whereas CD68-positive macrophages and CD66b-positive neutrophils were not dominant. These findings support CD8-positive T-cell–dominant myocardial injury with cytotoxic activation at the time of clinical myocarditis onset.

The pathological significance of this case lies in the shared CD8-positive T-cell–dominant inflammatory pattern across multiple irAE-associated organs. Although terminal septic shock and circulatory failure were present at autopsy and likely contributed to some findings, such as hepatic congestion and acute tubular necrosis, CD3-positive and CD8-positive T-cell infiltration was observed in the myocardium, pancreas, salivary gland, pharynx/larynx, kidney, and intestine. Quantitative multiplex immunofluorescence analysis further demonstrated that CD8-positive T cells were enriched in irAE-associated organs and tissue sites, whereas CD8-positive T-cell infiltration was limited in the sampled hepatic tumor and background liver. This distribution suggests that the inflammatory infiltrates were not merely a nonspecific systemic terminal phenomenon but were preferentially associated with immune-mediated organ injury.

Granzyme B expression was detected in a subset of CD8-positive T cells but varied among organs. Therefore, the overall pattern should be interpreted as CD8-positive T-cell–dominant immune-mediated injury with variable cytotoxic activation, rather than uniform Granzyme B-positive cytotoxic T-cell infiltration across all organs. In contrast, CD68-positive macrophages were scattered, and CD66b-positive neutrophils were sparse. Abscess formation or neutrophil-dominant suppurative inflammation was not evident. These findings support systemic T-cell–mediated immune injury rather than terminal nonspecific inflammation or neutrophil-dominant infectious inflammation. However, kidney involvement should be interpreted cautiously because acute tubular necrosis was also present; thus, immune-mediated tubulointerstitial injury may have coexisted with terminal circulatory injury rather than representing pure irAE nephritis. FOXP3 mini-panel staining showed that FOXP3-positive cells were relatively sparse compared with the prominent CD8-positive T-cell infiltration. This finding may support a CD8-dominant inflammatory milieu, but it does not directly prove regulatory T-cell depletion because pretreatment tissue was unavailable.

The relationship between immune activation and antitumor efficacy should also be interpreted cautiously (14). In the present case, an evident therapeutic effect was not confirmed in the hepatic tumor. Although irAEs have been suggested to correlate with antitumor efficacy in some settings, this relationship is complex and may be influenced by tumor type, treatment regimen, irAE phenotype, and corticosteroid therapy. Therefore, a direct causal relationship between severe irAEs and antitumor efficacy cannot be concluded from a single case.

This case has several limitations. First, this is a single case report and cannot represent the general pathophysiology of multisystem irAEs after STRIDE therapy. Second, terminal septic shock and circulatory failure were present at death, and their influence on some findings, particularly hepatic congestion and acute tubular necrosis, cannot be completely excluded. Third, most autopsy tissues were evaluated after corticosteroid therapy, which may have modified the degree and composition of inflammatory infiltrates. Therefore, the immune-cell proportions observed at autopsy may underestimate the intensity of inflammation during the active phase of each irAE. In addition, Granzyme B expression should be interpreted as evidence of variable cytotoxic activation among CD8-positive T cells, not as proof that all infiltrating CD8-positive T cells were uniformly cytotoxic. Fourth, T-cell receptor clonality analysis and single-cell RNA sequencing were not performed; therefore, clonality or shared antigen specificity of infiltrating T cells across organs could not be demonstrated. Nevertheless, the clear temporal relationship after STRIDE therapy, biopsy-proven myocarditis, organ-wide CD8-positive T-cell infiltration at autopsy, and multiplex immunofluorescence findings support systemic T-cell–mediated immune injury.

This case provides an important clinical lesson for the management of severe irAEs after STRIDE therapy. Improvement of one severe irAE does not necessarily indicate resolution of systemic immune activation. In the present case, myocarditis progressed metachronously after improvement of pancreatitis and upper-airway involvement. Therefore, after severe irAE onset, continued multisystem monitoring is important, particularly for delayed myocarditis and clinically silent organ involvement. Serial assessment of electrocardiography, cardiac troponin, BNP or NT-proBNP, echocardiography, pancreatic enzymes, renal function, urinalysis, gastrointestinal symptoms, upper-airway symptoms, and blood cell counts should be considered. In addition, although high-dose corticosteroid therapy is essential for life-threatening irAEs, infection surveillance and source control are also critical because intensive immunosuppression may increase infectious risk (15, 16). The fatal septic shock in this case underscores the difficulty of balancing irAE control with infection management.

In conclusion, this case demonstrates that severe irAEs after tremelimumab plus durvalumab therapy may progress metachronously and extend to clinically silent organs as systemic CD8-positive T-cell–dominant immune-mediated injury. Clinically apparent irAEs may represent only the visible portion of systemic immune-mediated injury, and continued multisystem monitoring is warranted after severe irAE onset.

## Data Availability

The original contributions presented in the study are included in the article/**Supplementary Material**. Further inquiries can be directed to the corresponding authors.
