# Supplementary material for: Metachronous severe multisystem immune-related adverse events during tremelimumab plus durvalumab therapy for hepatocellular carcinoma: a clinicopathological autopsy case report
Source: Front Oncol. 2026 Aug 5;16:1830325. doi: 10.3389/fonc.2026.1830325 (PMC13485758; doi:10.3389/fonc.2026.1830325)
Supplement: Supplementary file 1 [file Table1.docx]

**Supplementary Figure 1. Individual-channel multiplex immunofluorescence images of immune-related multiorgan injury.**
(A) Individual-channel images corresponding to the multiplex immunofluorescence findings shown in Figure 3 are presented for representative irAE-associated autopsy tissues. The Opal panel included DAPI, CD3, CD4, CD8, Granzyme B, CD66b, and CD68. Merged images and each individual marker channel are shown in the same organ order as in Figure 3.
(B) Individual-channel images of the FOXP3 mini-panel are shown for the corresponding tissues. The panel included DAPI, CD4, and FOXP3. These images support the interpretation that CD8-positive T-cell infiltration was prominent in multiple irAE-associated organs, whereas CD68-positive macrophages, CD66b-positive neutrophils, and FOXP3-positive CD4-positive cells were not the dominant inflammatory populations. Scale bar: 100 µm.

**Supplementary Figure 2. Multiplex immunofluorescence findings in hepatic tumor and background liver.**
(A) Multiplex immunofluorescence images of the sampled hepatic tumor and background liver using the Opal immune-cell panel, including DAPI, CD3, CD4, CD8, Granzyme B, CD66b, and CD68. Merged images and individual marker channels are shown.
(B) Multiplex immunofluorescence images of the FOXP3 mini-panel in the sampled hepatic tumor and background liver. The panel included DAPI, CD4, and FOXP3.
(C) Quantitative analysis of immune-cell subsets in the sampled hepatic tumor and background liver. CD8-positive T cells were limited in both regions compared with irAE-associated organs. These comparator findings support the interpretation that CD8-positive T-cell infiltration was preferentially enriched in immune-related organ injury rather than uniformly increased across all sampled tissues. Percentages indicate the proportion of each immune-cell subset among total nucleated cells in the analyzed regions. Scale bar: 100 µm.

Supplementary Table 1. Organ-based clinicopathological summary of immune-related multiorgan injury

| **Organ/site** | **Clinical presentation** | | **Histopathological findings** | **Multiplex immunofluorescence phenotype** | | **Interpretation** |
| --- | --- | --- | --- | --- | --- | --- |
| **Clinically apparent irAE-affected organs** | | |  |  | |  |
| Pancreas | | Grade 3 pancreatitis; pancreatic enlargement on CT | Peripancreatic fat necrosis, lobular fibrosis, and acinar atrophy | CD8+ T cells accounted for 26.7% of total nucleated cells, with a relatively high CD8+GZMB+ fraction. FOXP3+CD4+ cells were sparse, and CD68+ and CD66b+ cells were not dominant. | CD8+ T-cell–dominant immune-mediated pancreatic injury with cytotoxic activation | |
| Salivary gland | | Salivary gland enlargement; clinically diagnosed sialadenitis | Periductal fibrosis and lymphocytic infiltration | CD8+ T cells accounted for 15.1% of total nucleated cells. CD8+GZMB+ and CD8+GZMB− subsets were both present. FOXP3+CD4+ cells were sparse, and CD68+ and CD66b+ cells were not dominant. | Immune-related sialadenitis with CD8+ T-cell–predominant inflammation | |
| Pharynx/larynx/hypopharynx | | Grade 4 laryngeal edema; upper-airway stenosis requiring intubation | Residual hypopharyngeal edema with subepithelial and intraepithelial lymphocytic infiltration | CD8+ T cells accounted for 15.3% of total nucleated cells in the larynx and 45.1% in the pharynx. In the pharynx, CD8+GZMB− T cells were the major CD8+ subset. CD66b+ neutrophils were present but not dominant. | Persistent immune-mediated upper-airway inflammation with prominent CD8+ T-cell infiltration | |
| Endomyocardial biopsy | | Wenckebach-type AV block progressing to complete AV block; troponin I 42,006 pg/mL; decreased LVEF | Myocyte injury, focal myocardial dropout, and interstitial inflammatory infiltration | CD8+ T cells accounted for 62.7% of total nucleated cells. Both CD8+GZMB+ and CD8+GZMB− T cells were present, with CD8+GZMB− cells representing the larger subset. CD68+ and CD66b+ cells were not dominant. | Biopsy-proven CD8+ T-cell–dominant ICI myocarditis | |
| Autopsy myocardium | | Clinically diagnosed myocarditis after corticosteroid therapy | Patchy fibrosis and residual lymphocytic infiltration | CD8+ T cells accounted for 15.4% of total nucleated cells. Residual CD8+GZMB+ and CD8+GZMB− T cells were detected, whereas CD68+ and CD66b+ cells were not dominant. | Persistent/resolving CD8+ T-cell–dominant myocarditis | |
| **Clinically silent organ involvement identified at autopsy** | | | |  | |  |
| Kidney | | No prominent clinical diagnosis of renal irAE; terminal septic shock/circulatory failure present | Acute tubular necrosis with mild interstitial lymphocytic infiltration | CD8+ T cells accounted for 31.4% of total nucleated cells. CD8+GZMB− T cells were the major CD8+ subset, whereas CD8+GZMB+ cells were limited. CD66b+ neutrophils were not dominant. | Possible immune-mediated tubulointerstitial injury superimposed on terminal acute tubular injury | |
| Small intestine/ileum | | No prominent enteritis, severe diarrhea, or overt gastrointestinal bleeding during life | Mucosal hemorrhage and lymphocytic infiltration in the lamina propria and residual epithelium | CD8+ T cells accounted for 26.2% of total nucleated cells. CD8+GZMB− T cells were the major CD8+ subset, whereas CD8+GZMB+ cells were limited. CD66b+ neutrophils were not dominant. | Clinically silent immune-mediated small intestinal involvement | |
| **Comparator tissues** | | |  |  | |  |
| Liver tumor | | No evident radiological antitumor response | Viable hepatocellular carcinoma tissue | CD8+ T cells were limited, accounting for 1.8% of total nucleated cells. CD66b+ cells were relatively more frequent than CD8+ T cells in the sampled tumor area. | No prominent CD8+ T-cell–dominant antitumor immune infiltration in the sampled tumor tissue | |
| Background liver | | No clinically apparent immune-mediated hepatitis | Non-tumorous liver tissue | CD8+ T cells were limited, accounting for 5.4% of total nucleated cells. The inflammatory profile was not CD8+ T-cell dominant. | No prominent CD8+ T-cell–dominant immune-mediated hepatitis in the sampled background liver | |

Abbreviations: AV, atrioventricular; CT, computed tomography; GZMB, granzyme B; ICI, immune checkpoint inhibitor; irAE, immune-related adverse event; LVEF, left ventricular ejection fraction.

Notes: CD8+ T cells were defined as CD3+CD8+ cells. CD8+GZMB+ T cells were defined as CD3+CD8+GZMB+ cells, and CD8+GZMB− T cells were defined as CD3+CD8+GZMB− cells. FOXP3+CD4+ cells were defined as CD3+CD4+FOXP3+ cells. Percentages indicate the proportion of total nucleated cells in the analyzed regions. GZMB expression among CD8+ T cells varied among organs; therefore, the overall pattern was interpreted as CD8+ T-cell–dominant immune-mediated injury with variable cytotoxic activation, rather than uniform GZMB-positive cytotoxic T-cell infiltration across all organs.
